# Polyurethane Shape Memory Polymer/pH-Responsive Hydrogel Hybrid for Bi-Function Synergistic Actuations

**DOI:** 10.3390/gels9050428

**Published:** 2023-05-21

**Authors:** Shuyi Peng, Xingyu Cao, Ye Sun, Lin Chen, Chao Ma, Lang Yang, Hongliang Zhao, Qijie Liu, Zhenzhong Liu, Chunxin Ma

**Affiliations:** 1 State Key Laboratory of Marine Resource Utilization in South China Sea, Hainan University, Haikou 570228, China; 20080500210021@hainanu.edu.cn (S.P.); 20085600210002@hainanu.edu.cn (X.C.); sunye@hainanu.edu.cn (Y.S.); 22110805000014@hainanu.edu.cn (L.C.); 20080500210019@hainanu.edu.cn (C.M.); yl13339833255@163.com (L.Y.); 2 Key Laboratory of Quality Safe Evaluation and Research of Degradable Material for State Market Regulation, Products Quality Supervision and Testing Institute of Hainan Province, Haikou 570203, China; 3 Taizhou Key Laboratory of Medical Devices and Advanced Materials, Research Institute of Zhejiang University-Taizhou, Taizhou 318000, China; tanksman@163.com (Q.L.); zzliu@zju.edu.cn (Z.L.)

**Keywords:** stimuli-responsive hydrogels, shape memory polymers, actuations, programmability, bio-mimetics

## Abstract

Stimuli-responsive actuating hydrogels response to the external stimulus with complex deformation behaviors based on the programmable anisotropic structure design are one of the most important smart soft materials, which have great potential applications in artificial muscles, smart values, and mini-robots. However, the anisotropic structure of one actuating hydrogel can only be programmed one time, which can only provide single actuating performance, and subsequently, has severely limited their further applications. Herein, we have explored a novel SMP/hydrogel hybrid actuator through combining polyurethane shape memory polymer (PU SMP) layer and pH-responsive polyacrylic-acid (PAA) hydrogel layer by a napkin with UV-adhesive. Owing to both the super-hydrophilicity and super-lipophilicity of the cellulose-fiber based napkin, the SMP and the hydrogel can be bonded firmly by the UV-adhesive in the napkin. More importantly, this bilayer hybrid 2D sheet can be programmed by designing a different temporary shape in heat water which can be fixed easily in cool water to achieve various fixed shapes. This hybrid with a fixed temporary shape can achieve complex actuating performance based on the bi-functional synergy of temperature-triggered SMP and pH-responsive hydrogel. The relatively high modulus PU SMP achieved high to 87.19% and 88.92% shape-fixing ratio, respectively, correspond to bending and folding shapes. The hybrid actuator can actuate with the 25.71 °/min actuating speed. Most importantly, one SMP/hydrogel bi-layer hybrid sheet was repeatedly programmed at least nine times in our research to fix various temporary 1D, 2D and 3D shapes, including bending, folding and spiraling shapes. As a result, only one SMP/hydrogel hybrid can provide various complex stimuli-responsive actuations, including the reversable bending-straightening, spiraling-unspiraling. A few of the intelligent devices have been designed to simulate the movement of the natural organisms, such as bio-mimetic “paw”, “pangolin” and “octopus”. This work has developed a new SMP/hydrogel hybrid with excellent multi-repeatable (≥9 times) programmability for high-level complex actuations, including the 1D to 2D bending and the 2D to 3D spiraling actuations, which also provides a new strategy to design other new soft intelligent materials and systems.

## 1. Introduction

Nowadays, hydrogel actuators [1,2,3] have great development and potential applications in artificial muscle [4], grippers [5], soft robots [6], etc., on account of the soft and biocompatibility of the hydrogel. The hydrogel actuator can respond to single/multiple stimulus such as pH [7,8], ionic strength [9,10], temperature [11,12,13], light [14,15], electricity [16] and magnetism [17]. The multiple stimuli-responsive [6] ability enables actuators more extensive applications, while the simple deformation behaviors can limit the development. To improve the deformation complexity of the actuator, various anisotropic structures (stratified structure [18,19,20], gradient structure [21,22], orientation structure [23], patterned design [24] and 3D printing [25,26]) was applied to construct the actuator for achieving more and complex deformations.

In general, hydrogels [27] were natural isotropics, which only had the volume changes of swelling and shrinking with the response to external stimulus. The construction of anisotropic structure became a common way to achieve 2D/3D deformation [28,29,30]. Zhang et al. [31] achieved the actuator bending/folding and imitated as the octopus-inspired soft swimmer by the combination the composite hydrogel with poly(dimethylsiloxane) with low coefficient of expansion. Shojaeifard et al. [32] had constructed a bi-layer actuator with a different pH-responsive, which can have bidirectional bending and reversible behaviors under the aqueous bath altering. Nevertheless, the programmable design about the original shape of the actuator based on the structure fabrication before response which can promote the hydrogel actuator behavior development. For example, Yin et al. [33] had achieved the different complex spiral deformation by the programmable patterned shape design which was based on the fabrication stage, but the actuating behaviors can reply on the original network structure, which need a lot of time to prepare. Dai et al. [34] fixed the magneto orientation of magnetic double stacks (MDSs) in hydrogel by programmable the rotating magnetic field, which can cause the complex structure and various deformations. However, once an actuator was prepared, its original shape and the subsequent deforming performance was determined already. Therefore, these hydrogel actuators cannot have multiple actuating modes at the same hydrogel.

The single actuating mode of the hydrogel actuator (the designed structure has the only corresponding deformation type) cannot achieve that. The same actuator has multiple complex actuating behaviors, which limited more widespread application. Therefore, in order to fit the requirement of complex applications, it is necessary to design a novel actuator with multiple complex actuating modes to expand the actuating behaviors. Focusing on the problem, the combination of the shape memory property and stimuli-responsive achieved the complex actuating behaviors under multiple shapes, which provide an idea for solving the single actuating modes of the actuator. Chen et al. [35] composited the shape memory hydrogel and actuating hydrogel forming a bi-layer structure actuator which can achieve response to external stimulus under various shapes. However, on account of the low modulus shape memory hydrogel, the shape fixing rate needs to be improved and the shape fixed, by way of the hydrogel, by a metal coordination reaction which was complicated.

Shape memory polymer (SMP) [36,37,38], as one of the intelligent response materials which contained the shape memory hydrogel, can respond to external stimulus, such as high temperature [39]. The common SMPs were not the hydrogel-based, which were mainly composed of highly crosslinked polymer networks, such as polyethylene-based [40], polyester [41], polyurethane polymers [42,43], etc. Yang et al. fabricated a two-way shape memory polyurethane as a middle layer which was stuck in nanofibrous polyimide (PI) film, and the microfibrous nylon fabric formed the thermal-triggered intelligent switch and was designed as a movable triboelectric nanogenerator. Compared with the shape memory hydrogel, the typical SMP had a higher modulus, which caused a high fixing rate [44,45]. The SMP have the advantage of fixing the temporary shapes by heating. However, most SMPs (like the epoxy resin) had the relatively high elastic modulus, which can reach over 10 MPa of the elastic modulus and cannot firmly combine with relatively soft hydrogels [46,47]. The polyurethane shape memory polymer (PU SMP) is relatively soft [48,49] and can reach less than 2 MPa of the elastic modulus [50,51], which can help several of the soft hydrogels achieve a high shape fixing ratio and can be relatively soft to combine with a soft hydrogel firmly. Meanwhile, the pH-responsive hydrogel cannot influence the temperature-triggered PU SMP to design various temporary shapes. Therefore, the PU SMP can combine with the pH responsive hydrogel to design a novel actuator with dual-function synergy to achieve complex actuations.

**Scheme 1:**
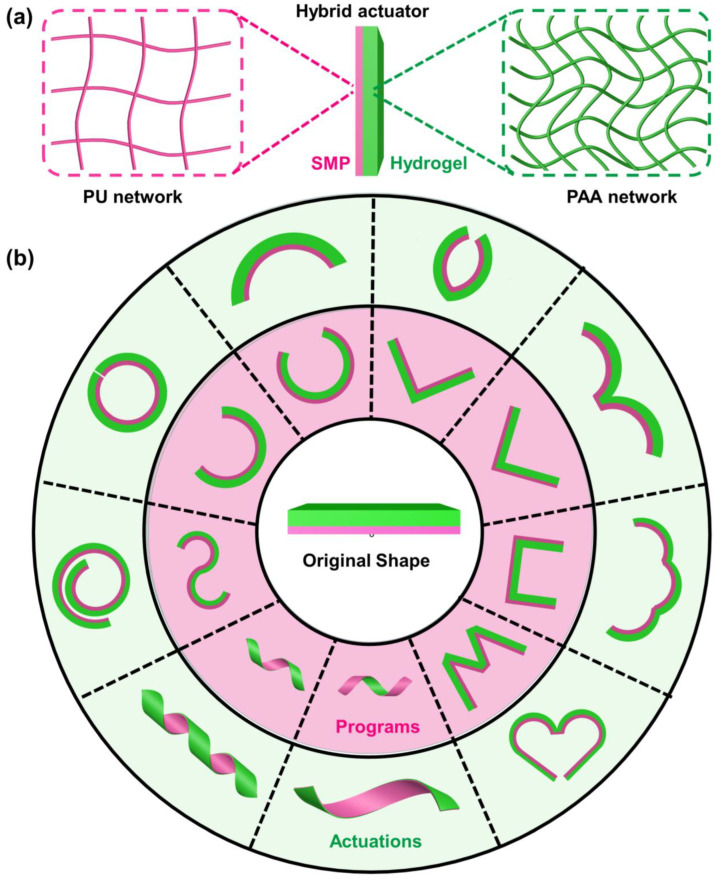
Schematic illustration of (**a**) structure of the hybrid actuator and (**b**) the actuations of the hybrid under programmable designed shapes.

Herein, we present a novel hybrid actuating material which we combined with a temperature-triggered polyurethane shape memory polymer (PU SMP) layer and a pH-responsive polyacrylic-acid (PAA) hydrogel layer (Scheme 1a). Owing to both the super-hydrophilicity and super-lipophilicity of the cellulose- based napkin, the SMP and the hydrogel can be bonded firmly by the UV-adhesive added in the napkin. This hybrid 2D sheet can be programmed by designing a different temporary shape in heated water, which can be fixed easily in cool water to achieve various fixed shapes. This hybrid with a fixed temporary shape can achieve complex actuating performance based on the bi-functional synergy of temperature-triggered SMP and pH-responsive hydrogel. Most importantly, one SMP/hydrogel bi-layer hybrid sheet can be repeatedly programmed many times for various fixed temporary 1D, 2D and 3D shapes and subsequently different complex actuations (Scheme 1b). Therefore, only one SMP/hydrogel hybrid can provide various complex stimuli-responsive actuations, which can be utilized to design various intelligent devices and will extend the applications of the stimuli-responsive deformational hydrogel actuator.

## 2. Results and Discussion

### 2.1. Fabrication and Characteristic of PAA Hydrogel and Polyurethane SMP

**Figure 1:**
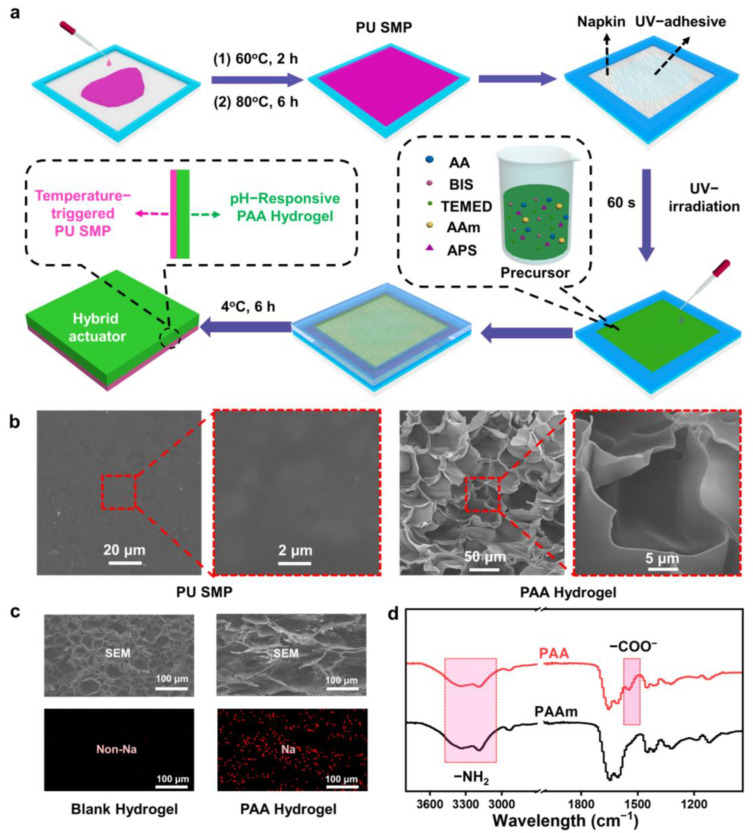
(**a**) The preparation diagram of the hybrid actuator. (**b**) SEM images of PAA hydrogel and polyurethane SMP. (**c**) Comparison of EDS-mapping images between blank hydrogel and PAA hydrogel. (**d**) The FTIR spectra of PAAm (blank hydrogel) and PAA hydrogel.

After the preparation of the polyurethane shape memory polymer (PU SMP) under the thermosetting, the cellulose-fiber based napkin and polyurethane SMP were bonded by curing the UV-adhesive. The polymerization of the polyacrylenic acid (PAA) hydrogel was on the napkin surface to form a bi-layer structure, which included a thermal-triggered SMP layer and a pH-responsive hydrogel layer, as shown in Figure 1a. The smooth polyurethane SMP network was significantly compacted and had no holes, according to Figure 1b. In contrast to the smooth PU SMP, the PAA hydrogel had porous construction; the network and the thin wall of the variable-sized hole can endow the hydrogel high swelling property. As shown in Figure 1c, comparing the Na element distribution of the PAA hydrogel and the blank hydrogel, it can be seen that the Na element was uniformly dispersive in the hydrogel network, which proved the methacrylic acid (AA) was randomly combined with acrylamide (AAm) to form a copolymer. The FT-IR spectrogram in Figure 1d also confirmed that the PAA hydrogel was successfully constructed. The wide peak at 3100–3500 cm^−1^ was indicated to -NH_2_ from PAAm. Meanwhile, the –COO^−^ groups were observed at 1550 cm^−1^, which were the characteristic peaks of PAA hydrogel. The above test can completely demonstrate that the pH-responsive hydrogel was successfully constructed.

**Figure 2:**
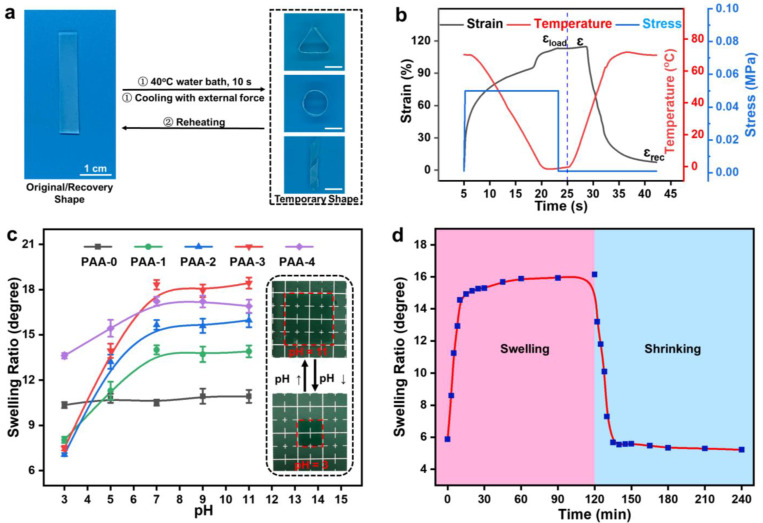
(**a**) The shape fixing diagram of the polyurethane SMP. (**b**) The fixing rate of the polyurethane SMP. (**c**) The swelling ratio of different content AA hydrogels in the gradient increasing pH buffer solutions (IS = 0.1 mol/L). (**d**) The dynamic curve of the PAA-3 hydrogel swelling and shrinking.

The PU SMP also had a great shape memory property, which was significantly important for the bi-function synergistic actuator. As shown in Figure 2a, the same PU SMP strip can be designed as different shapes, such as triangle, circle and spiral. The critical temperature of the thermos-triggered PU SMP was 37 °C (Figure S1); the PU SMP can become soft under the temperature over 37 °C. Therefore, the temporary shapes fixing process was that the PU SMP immersed in 40 °C water bath for 10 s, and the soft PU SMP can pose as its temporary shape with external force and cooling to fix it. It was easy to repose the designed temporary shapes by reheating above the critical temperature. Meanwhile, the DMA test obviously revealed the high shape fixing rate (R_f_) and recovery rate (R_r_), which were 99.82% and 99.32%, respectively (Figure 2b). R_f_ and R_r_ were calculated by the Equations (1) and (2) as follows:

(1) $$R_{f}=\frac{\varepsilon }{\varepsilon_{load}}\times 100\%$$

(2) $$R_{r}=\frac{\varepsilon -\varepsilon_{rec}}{\varepsilon }\times 100\%$$

where ε_load_ is the maximal strain under constant 0.05 MPa external force and ε is the strain after removing external force. ε_rec_ is the strain that the sample finished recovery without external forces under the shape memory recovery temperature.

Besides the digital R_f_ and R_r_ from the DMA test, it also can be expressed by the comparison of the angle of the designed temporary shape (θ_1_) and fixed shape (θ_2_). The recovery angle was recorded as θ_3_ (Figure S2). The R_f_ and R_r_ were calculated by the Equations (3) and (4) as follows:

(3) $$R_{f}=1-│\frac{\theta_{1}-\theta_{2}}{\theta_{1}}│\times 100\%$$

(4) $$R_{r}=\frac{\theta_{3}}{180}\times 100\%$$

The R_f_ of the PU SMP was near 99.8% by measuring the angle’s difference of designed and fixed shape (Figure S3). Meanwhile, the shape memory property of the PU SMP can not reduce much after five times cycles. The R_f_ can still remain above 96%, while the R_r_ can slightly decline to 91% (Figure S4).

The pH response ability of the actuator was from the ionization level of carboxylic acid groups in AA, which can determine the actuations. When the PAA hydrogel was in acidic condition and whose pH was lower than the acidity coefficient of the carboxylic acid (pKa(–COOH) ≈ 4.3), the carboxyl group can exist as –COOH, and the hydrogel can shrink. While the carboxyl group can become dissociated and exist as –COO^−^ in the solution whose pH was over 4.3, then the hydrogel swelling occurred.

For more powerful actuation force to drive the polyurethane SMP to move, we studied on the influence of the different AA content on the hydrogel volume change. The different content (0.5 wt%, 1.0 wt%, 1.5 wt%, 2 wt%) AA was added to form composite hydrogel which were named as PAA-1 to PAA-4. The blank hydrogel (PAA-0) was pure PAAm hydrogel without any AA. As shown in Figure 2c, PAA-0 hydrogel was insensitive for the change of pH; its swelling ratio (SR) was gentle, while the PAA hydrogels were obviously changing with the pH increase. The SR was gradually increasing and tending to saturation when the pH was over 7. Observing the SR difference between pH = 3 and pH = 11 buffer solution (IS = 0.1 mol/L), it was the maximum when the AA content was 1.5 wt%. The SR was increasing with the AA content increasing in pH = 11, but it can decrease while the AA content is above 1.5 wt%, as shown in Figure S5. The reduced SR and difference may be due to the extreme AA content and can cause the cross-linking density and electrostatic repulsion increase. The optimal proportion for great pH response was the PAA-3, which represented 1.5 wt% content AA hydrogel; its SR difference was 10.95. From the images of PAA-3 in pH = 3 and pH = 11, there was the remarkable size change; the length in pH = 11 was almost double compared to pH = 3. The volume change of the PAA-3 hydrogel from pH = 3 to pH = 11 was nearly quadruple (Figure 2c). Moreover, the kinetic curve in Figure 2d showed that the swelling process reaching equilibrium needed 30 min, and the fast swelling was the first 10 min which was almost 90%, while the shrinking process can need 30 min to totally shrink.

### 2.2. Fabrication and Characteristic of the Actuator with Bi-Function Synergy

**Figure 3:**
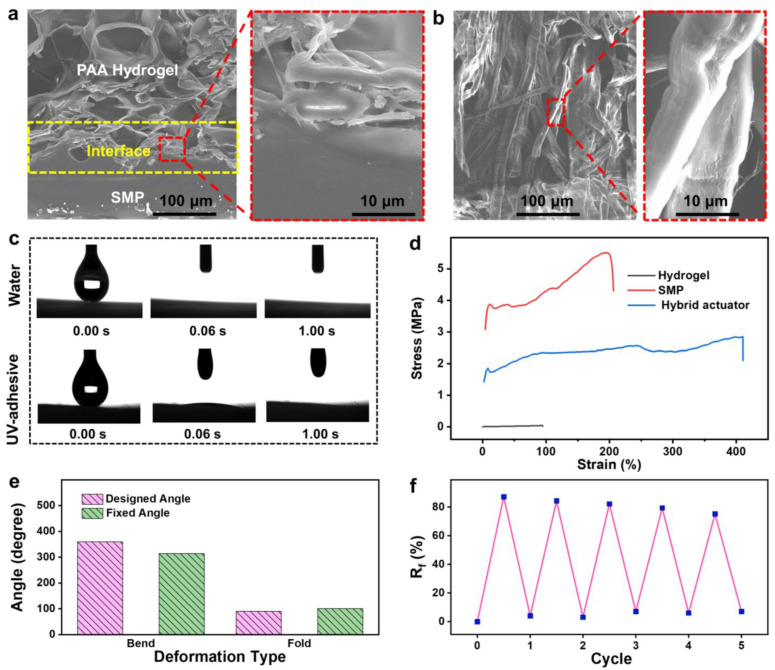
(**a**) SEM images of the actuator cross section (**b**) The SEM images of the cellulose-fiber based napkin. (**c**) The contact angle of the cellulose-fiber based napkin for water and UV glue. (**d**) The mechanical property of the PAA hydrogel, PU SMP and the hybrid actuator. (**e**) The fixing rate of the actuator. (**f**) The shape fixing cycle test of the actuator.

Once hydrogel in situ polymerized on the PU SMP surface, the actuator can separate easily after being put into water. Therefore, the hydrophilia and lipophilicity cellulose-fiber based napkin became a choice as the interlayer by using UV-adhesive to combine PU SMP and as the carrier for bonding hydrogel to solve the separation problem. As shown in Figure 3b and Figure S6, the optical images and microstructure of the cellulose-fiber based napkin illustrated that it had a loose network structure. The cellulose diameter was 10 ± 5 μm and the holes enabled it to absorb the liquid quickly. The cellulose-fiber based napkin can absorb and disperse the water quickly; the contact angle shows as 0° at 0.06 s. It was also excellent for absorbing the UV-adhesive, which was acrylic ester as its main component; the contact angle was 16.4° at 0.06 s and can totally disperse in the napkin after 1.00 s (Figure 3c). Therefore, we successfully fabricated the bi-layer actuator by using the cellulose-fiber based napkin as shown in Figure 3a. The UV-adhesive cured on the surface of PU SMP to realize the firm bonding, and the PAA hydrogel immersed in the cellulose-fiber based napkin which can form a strong mechanical interlocking force. There were many hydrogen bonds formed between the PAA hydrogel and the cellulose-fiber based napkin which caused stronger bonding. Therefore, we solved the problem of interface bonding and gained the anisotropic actuator with bi-layer structure.

The introduction of PU SMP into the actuator system and firm combination with hydrogel can significantly improve the mechanical strength. Comparing the mechanical properties among the PU SMP, PAA hydrogel and the hybrid actuator as shown in Figure 3d, the high tensile strength of the PU SMP (5.4 ± 0.3 MPa) can endow the actuator a high mechanical property, which was 2.5 ± 0.3 MPa, while the PAA hydrogel was just only 39 ± 3 KPa in Figure S7, which revealed that the tensile strength was greatly improved by 64.1 times. Meanwhile, the bi-layer structure and wet condition of the hybrid actuator caused elongation at the break and was nearly twice the number of times as PU SMP.

The higher modulus of the PU SMP (1.2 MPa) compared with shape memory hydrogel had a higher fixing rate. The temporary shape fixing process of the hybrid actuator was the same as PU SMP; it was fixed with external force after 30 s in hot water (40 °C), and the angle of the actuator at this time was recorded as θ_1_. Then, the actuator with the shape fixed was recorded as θ_2_. The R_f_ was calculated by Equation (3). We tested the fixing rate of the actuator for the temporary shape of circle and right-angle; the angles of the actuator designed and fixed were shown in Figure S8. Compared with the changes of the angles, the R_f_ were 87.19% and 88.92% corresponding to the shape of bending and folding (Figure 3e). Such excellent shape memory property endowed the hybrid actuator the high controllability of the temporary shapes, which realized that the actuator had more complex temporary shapes and actuations for meeting the shape design requirements. In addition, the cycle test of the hybrid actuator was also tested for five times, and the R_f_ remained about 73%, which is significant high (Figure 3f). The decrease of the R_f_ after several programmability times may be because the hybrid actuator worked at 25 °C, which was relatively close to the critical shape transformation temperature of the PU SMP (37 °C). That is, after repeatedly being programmed for many times, the R_f_ of the hybrid actuator can decrease due to the partial recovery of the PU SMP polymeric chain from the temporary shape at 25 °C.

### 2.3. pH-Responsive Actuations of the Actuator

**Figure 4:**
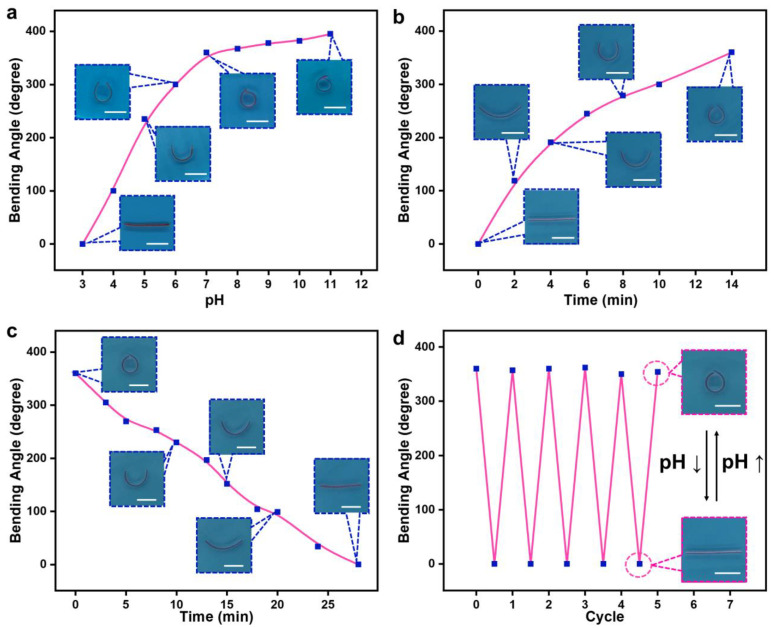
(**a**) The bending angle of the actuator in different pH buffer solutions (IS = 0.1 mol/L). (**b**) The swelling process of the actuator in pH = 11 buffer solutions (IS = 0.1 mol/L). (**c**) The recovery process of the actuator in pH = 3 buffer solutions (IS = 0.1 mol/L). (**d**) The cycle test of the actuator under different pH buffer solutions (IS = 0.1 mol/L). All scale bars are 10 mm.

The actuator can respond to the pH change because of the swelling behavior of the PAA hydrogel, which can drive the PU SMP movement. As shown in Figure 4a, we explored the final angles of the actuator under different pH solutions; it needed at least 2 h to fully swell, and we measured the angles as the final angles. The equilibrium angle in pH = 3 was 0°; therefore, it was determined as the original state of the actuator. The curve and images of the bending angles at equilibrium showed that it can increase with the pH growing. The angles of the hybrid actuator can become over 360° when the pH was over 7. Therefore, the actuation of the hybrid actuator was put into pH = 11, and the hydrogel began swelling to drive the original state actuator movement.

In addition, the actuation of the hybrid actuator was recorded in Figure 4b; the beginning angle of the actuator (10 × 1.5 × 0.3 mm^3^) was 0° in pH = 3 buffer solution, and the strip gradually closed. The angle became bigger due to the –COOH group in the PAA hydrogel forming the –COO^−^ group. The network of the PAA hydrogel was expanding to absorb water. After 14 min swelling, the angle of the hybrid actuator became 360°, with the 25.71 °/min actuation speed. The whole actuation process was the shape of the hybrid actuator change from the straight strip to circle. After the actuation in pH = 11, the hybrid actuator was put back to the pH = 3 for recovering. Comparing the actuation process, the shrinking speed was significantly lower, as shown in Figure 4c. The shrinking process was 28 min, and the average actuating speed was 12.86 °/min.

Moreover, the circulation test of the hybrid actuator revealed a nice durability. Under the five times test, Figure 4d showed the bending angle changes of the actuator, which were without large fluctuation, by continuously changing the solution from pH = 3 to pH = 11. Besides the actuating test of the hybrid actuator at room temperature (25 °C), we also measured the actuations in the cool water (5 °C) and physiological body warm temperature (37 °C). As shown in Figure S9, the actuating speed in the cool water (5 °C) decreased significantly compared with that at room temperature. It took nearly 1 h to actuation from a strip to a circle, which was about four times as long as that at room temperature. Meanwhile, in the physiological body-warm temperature water (37 °C), the hybrid actuator actuated as the following two stages (Figure S10). Firstly, the hybrid actuator with the programmatically designed “S” temporary shape recovered to a strip-shape (the original shape of the PU SMP) in the first 5 s, due to the physiological body warm temperature water (37 °C) reached the critical shape transformation temperature of the temperature-triggered PU SMP. Then, the hybrid strip-shape actuator gradually bent with faster speed than that at room temperature because of both the softening of the PU SMP and the faster pH-responsiveness at 37 °C of the PAA hydrogel than that at 25 °C. However, the hybrid actuator at physiological body temperature (37 °C) cannot remain in a various programmable temporary shape of the PU SMP, and it can only provide pH-responsive actuation from the original strip-shape.

### 2.4. The Actuation Behaviors and Biomimetic Applications of the Hybrid Actuator under Programmable Shape Design

**Figure 5:**
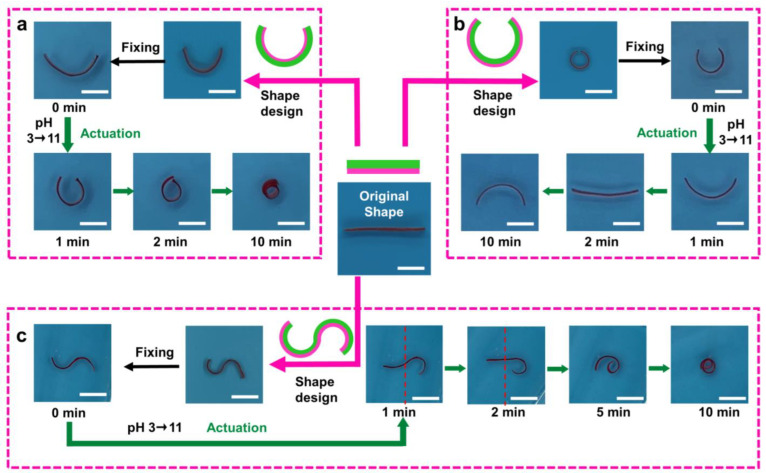
(**a**) The actuation of the designed arc shape actuator with hydrogel outside. (**b**) The actuation of the designed arc shape actuator with hydrogel inside. (**c**) The actuation of the designed arc shape actuator with hydrogel inside. All scale bars are 10 mm.

The relatively high modulus PU SMP endowed high shape controllability of the hybrid actuator. As shown in Figure 5a, the actuator with a straight original shape was programmable and designed as the arc shape, which was the hydrogel outside and PU SMP inside. It showed the actuation of the hybrid actuator in a pH = 11 buffer solution; it can bend toward the PU SMP on account of the hydrogel swelling and, finally, became a 540° circle shape through a 15 min swelling process. Contrary to the arc shape with the outside PU SMP, we reprogrammed the hybrid actuator as the hydrogel inside and the PU SMP outside the temporary shape and showed that it can actuate differently (Figure 5b). The designed hybrid actuator can first open the angle and become a straight strip in a temporary state and then bend to the reverse direction. The final shape of the designed actuator was the arc shape with the hydrogel outside through a 15 min swelling actuation, which realized the large angle reverse bending. Based on the different actuation process of these two differently designed shapes, we programmed the hybrid actuator as an “S” shape, which has the above two structures. As shown in Figure 5c, the actuation of the “S” shape actuator can be seen as the connection of two reverse directions arc shapes. At the first 3 min actuation of the “S” shape actuator, the right part actuated as the changes in Figure 5b while the left part moved, as the behavior in Figure 5a and Movie S1 shows. After the right part of the “S” actuator turned to another direction, it became actuating synergistically toward the center.

**Figure 6:**
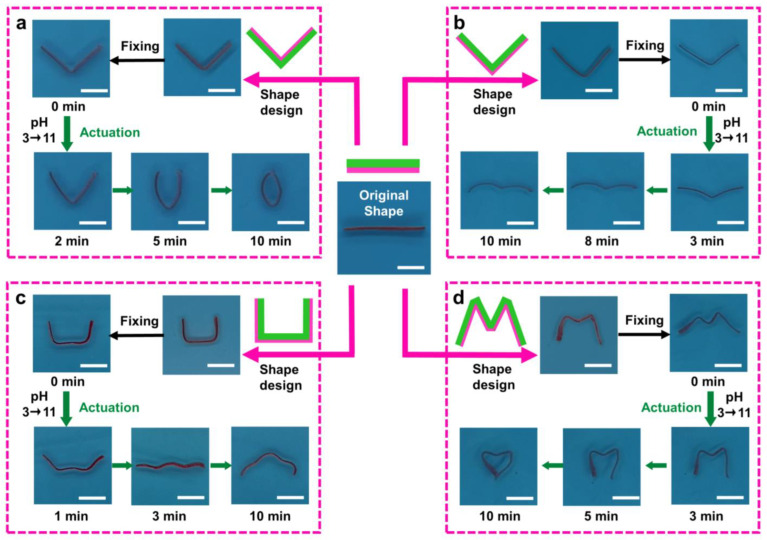
(**a**) The actuation of the designed right-angle shape actuator with hydrogel outside. (**b**) The actuation of the designed right-angle shape actuator with hydrogel inside. (**c**) The actuation of the designed “凵” shape actuator with hydrogel inside. (**d**) The actuation of the designed “M” shape actuator with hydrogel outside. All scale bars are 10 mm.

Beside various bending actuation of the hybrid actuator, it also can be designed with sharp angles. As shown in Figure 6a, the right angle with the hydrogel outside was designed, and the fixed angle had some changes, but the sharp connection was without great change. The actuation of the right-angle actuator can be seen as two straight strips bending and the connection closing. Because the PAA hydrogel became swollen, the actuation force pushed the soft PU SMP closed. Contrary to the right-angle shape actuator in which the hydrogel was outside, the hybrid actuator was reprogrammed as the hydrogel inside right-angle shape, and the actuation was totally different. With the hydrogel swelling, the inside hydrogel had outside force, and the straight strip also gradually bent. The images in Figure 6b show the opening process of the actuator and how it can form an obtuse-angle shape, becoming a shape like a sea gull whose wings were open through 10 min actuation process.

The strip actuator can be designed as a more complex shape with a sharp angle. The “凵” shape was designed, and it was fixed with more open “凵” shape. The designed shape with two right-angles with the hydrogel inside, the actuation of the “凵” shape, in fact, was the addition of the behavior in Figure 6b. As shown in Figure 6c, the actuation of the open “凵” shape followed as we designed, and it gradually become two sea gull shapes bonded. Because of the “_” position of the “凵” shape’s great bending movement, the final shape of the hybrid actuator became a cloud shape. Then, we reheated the actuator and reprogrammed it as an “M” shape with the hydrogel outside. The images in Figure 6d show the closing process of the “M” actuator; after 10 min actuation, the “M” gradually closed in the middle and became a “♡” shape (Movie S2).

**Figure 7:**
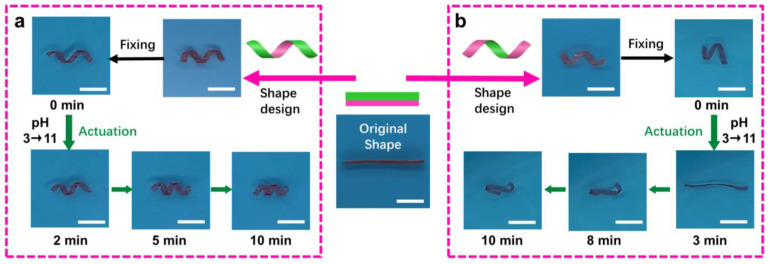
(**a**) The actuation of the designed spiral shape actuator with hydrogel outside. (**b**) The actuation of the designed spiral shape actuator with hydrogel inside. All scale bars are 10 mm.

On the basis of the actuation behavior in a 2D plane, the actuator also can fix the 3D shape to gain more complex deformation. As shown in Figure 7a, the spiral shape was designed with the hydrogel outside. The angle had some change between design and fixing, which was from 720° to around 540°. Because the hydrogel was outside, the actuation of the spiral shape was crimping toward the center line due to the pushing force. Comparing the images of the beginning and the final condition of the spiral actuator, the diameter and helical spacing of the actuator were significantly decreased. The angle at 540° increased to nearly 900°. In addition, the reversed direction spiral shape actuator with hydrogel inside can actuate differently. The spiral shape was designed, and the beginning shape angle of the hybrid actuator was about 480°. As shown in Figure 7b, the spiral actuator uncoiled and became a strip in the first 3 min. Then, the hybrid actuator screwed toward to the PU SMP (Movie S3). After 10 min actuation, the angle at this time was −300° because of the reversed direction.

The same straight strip actuator can have the basic bending actuation and can have the above 9 different types of actuations though the shape programmable designs, depending on the high shape memory property of the relatively high modulus PU SMP. Besides the above 10 actuations, the actuator can have other complex actuations by programmable shape design. Hereon, we achieved an actuator to have multiple actuating behaviors.

**Figure 8:**
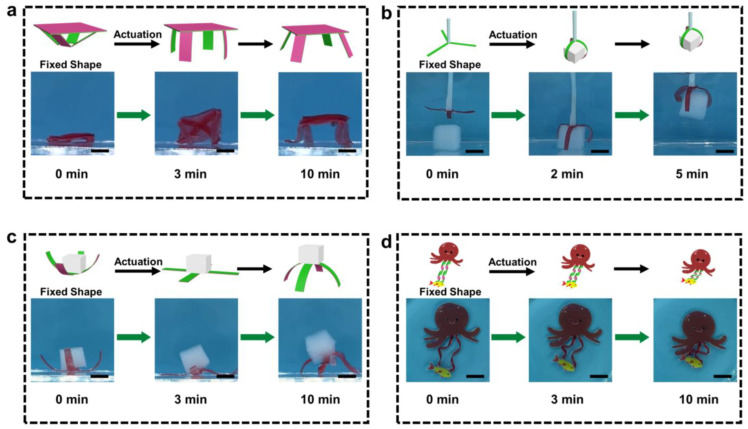
(**a**) The standing process of the folding table. (**b**) The catching process of the paw-mimetic actuator. (**c**) The jacking up process of the pangolin-mimetic actuator. (**d**) The hunting process of the octopus-mimetic actuator.

In order to prove that the bi-layer structure hybrid actuator has multiple applications, we designed the sheet actuator as a folding table. The temporary shape of the folding table was programmed and compressed as shown in Figure 8a and Movie S4. Because of the folding table designed as hydrogel inside, the legs can open toward the outside. The angles between table legs and board became bigger, and the whole table can stand up to become a table after fully swelling at last. The actuator also can be designed as a paw-mimetic actuator (paw) in which the PAA hydrogel was on top; the paw can begin bending and catch the targeted object whose weight was 538.6 mg, while the paw was 35.6 mg (Figure 8b and Movie S5). The paw can grab the object at least 15 times its own weight. Furthermore, a pangolin-mimetic actuator (pangolin) was designed, and the targeted object (1.034 g) was loaded, as shown in Figure 8c and Movie S6. Finally, the pangolin can down-bend and jack up the targeted object, which was about 20 times the actuator weight (52.6 mg). Moreover, it also can be designed as an octopus-mimetic actuator, which can imitate the octopus hunting process. According to Figure 8d and Movie S7, the legs of the octopus were the spiral actuator, and it can catch and move the fish close to the body of the octopus.

## 3. Conclusions

In summary, we have developed a shape memory polymer/hydrogel hybrid actuator with excellent bi-function synergyby combining a temperature-triggered polyurethane shape memory polymer (PU SMP) layer and a pH-responsive polyacrylic-acid (PAA) hydrogel layer by a napkin with UV-adhesive. The relatively high modulus PU SMP achieved the hybrid actuator high to 87.19% and 88.92% shape fixing ratio, respectively, correspond to bending and folding shapes, which can be repeatedly programmatically designed to any temporary shapes. Meanwhile, the hybrid actuator can actuate at the 25.71°/min actuating speed. Therefore, one SMP/hydrogel bi-layer hybrid can be repeatedly programmed at least 9 times for various fixed temporary 1D, 2D and 3D shapes and subsequently different complex actuations. As a result, only one SMP/hydrogel hybrid can provide various complex stimuli-responsive actuations, such as the reversable bending-straightening and spiraling-unspiraling. Furthermore, a few intelligent devices were designed to simulate the movement of the natural organisms, such as bio-mimetic “paw”, “pangolin” and “octopus”. This work provides a new SMP/hydrogel hybrid with excellent multi-repeatable (≥9 times) programmability for high-level complex actuations including the 1D to 2D bending and the 2D to 3D spiraling actuations, which also will inspire design of other new soft intelligent materials and systems.

## 4. Materials and Methods

### 4.1. Materials

Polycaprolactone diol (PCL, 99%), Polyhexamethylene diisocyanate (PHMD, 99%), Hexamethylene diisocyanate (HDI, 99%), Dibutyltin dilaurate (DBTDL, 99%), butyl ethanoate (99%), Acrylamide (AAm, 96%), methacrylic acid (AA, 98%), N,N′-Methylenebis(acrylamide) (BIS, 99%), Ammonium persulfate (APS), tetramethylethylenediamine (TEMED, 99%) and Ammonium peroxodisulfate (APS, 98%) were purchased from Macklin Inc., Rochelle, IL, USA. All the chemical reagents were used as received.

### 4.2. Polyurethane Shape Memory Polymer Preparation

The preparation method is based on ref. [51]. The polyurethane shape memory polymer (PU SMP) was prepared by two-stage thermal polymerization. First, 5 g PCL was melted in 100 °C vacuum oven, and we added 5 mL butyl acetate solvent to mix. The cross-linking agent PHMD and chain extender HDI (the functional group molar ratio was 1:9) were added into the mixture. Last, 0.5 wt% catalyst DBTD was added to speed up the chemical reaction. The mixture was thoroughly mixed under magnetic stirring and was poured on an aluminum sheet and spared evenly in the 0.1 mm silicon rubber mold to obtain PU SMP, whose thickness was around 100 μm. At last, the liquid mixture which limited in the 0.1 mm silicon rubber mold was cured in an oven at 60 °C for 2 h and dried in a vacuum oven at 80 °C overnight.

### 4.3. Bi-Function Synergistic Actuator Preparation

The preparation of the hybrid actuator was based on ref. [35]. First, PU SMP was prepared as Section 4.2. Second, applying UV-adhesive on the PU SMP, the cellulose-fiber based napkin, which was measured as about 30 μm, was placed upon the UV-adhesive and cured by UV irradiating. Then, preparing the hydrogel precursor, which was the mixture of 85 mg Aam, 15 mg AA, 4 mg BIS, 4 μL TEMED and 40 μL 4 wt% APS. The PAA precursor was polymerized on the cellulose-fiber based napkin in the 0.2 mm silicone rubber mold at 4 °C for 6 h. The 0.2 mm silicone rubber mold limited the PAA hydrogel thickness, which was around 200 μm. After the polymerization of the PAA hydrogel, the hybrid actuator was immersed in the 0.1 mol/L ionic strength PBS solution and cut as 20 × 3.5 × 1 mm^3^ for response behaviors measurements.

### 4.4. Characterization of Actutor

The morphology characterization and element distribution of the blank hydrogel, PAA hydrogel and the cross section of the actuator were observed by scanning electron microscope (SEM, HITACHI S-4800, Hitachi High Technologies, Ibaraki Prefecture, Japan). The component difference between blank hydrogel and PAA hydrogel was obtained by Fourier-transform infrared spectroscopic (FT-IR, Nicolet IS10, Thermo Electron Corporation, Waltham, Massachusetts, America). The above test samples needed to be freeze-dried to measure. The hydrophilia and lipophilicity of the cellulose-fiber based napkin was tested with a contact angle tester (JD2000D5, Powereach, Shanghai, China). The mechanical properties of the PAA hydrogel, PU SMP and the actuator were tested by a universal testing machine (GP-6113A, GAOPIN, Suzhou, China). The shape memory property of the PU SMP was tested by dynamic mechanical analysis (DMA Q800, TA Instruments, New Castle, Delaware, America). The DMA setting as the controlling force is 0.05 MPa; the temperature range is 0–70 °C, and the heating rate is 10 °C/min under the tensile mode. The pH adjustment was measured by pH meter (ST20, OHAUS, Parsippany, New Jersey, America). The actuating behaviors were photographed by iphone 13 pro, and the bending angle was measured using protractor software (Power Point, Version 2304).

### 4.5. Swelling Ratio Test of PAA Hydrogel

The equilibrium swelling ratio (SR) of PAA hydrogel in pH solution was tested by the same hydrogel. We prepared a hydrogel (30 × 30 × 0.2 mm^3^) and put into deionized water for fully swelling and then into different pH solutions (from pH = 3 to pH = 11) for at least 3 h. Therefore, the weights, after fully swelling in each of the pH solutions, were recorded as W_s_, and needed the water wiped from the hydrogel’s surface. Finally, freeze-dried hydrogel weight was recorded as W_d_. The SR was calculated by the following Equation (5).

(5) $$SR(\%)=\frac{Ws-Wd}{Wd}\times 100\%$$

## Data Availability

The data in this work are available from the corresponding author upon reasonable request.

## Supplementary Materials

The following supporting information can be downloaded at: https://www.mdpi.com/article/10.3390/gels9050428/s1, Figure S1: The DSC curve of the polyurethane SMP; Figure S2: The angel measurement diagram of the polyurethane SMP designed shape and fixed shape; Figure S3: The angels of the polyurethane SMP under designed shape and fixed shape; Figure S4: The cycle test of the polyurethane SMP; Figure S5: The swelling ratio of the different content PAA hydrogel in pH = 11 buffer solutions; Figure S6: The optional images of cellulose-fiber based napkin; Figure S7: The mechanical property of the PAA composite hydrogel; Figure S8: The Angles comparison of the actuator designed and fixed. Figure S9: The actuation of the strip actuator in pH = 11 solution at 5 °C. Figure S10: The actuation of the “S” shape actuator in pH = 11 solution at 37 °C. Movie S1. The actuation of the “S” shape actuator. Movie S2. The actuation of the “M” shape actuator. Movie S3. The actuation of the spiral shape actuator. Movie S4. The standing process of the folding table. Movie S5. The capturing process of the paw-mimetic actuator. Movie S6. The jacking up process of the pangolin-mimetic actuator. Movie S7. The hunting process of the octopus-mimetic actuator.
