# Supplementary material for: Polyurethane Shape Memory Polymer/pH-Responsive Hydrogel Hybrid for Bi-Function Synergistic Actuations
Source: Gels. 2023 May 21;9(5):428. doi: 10.3390/gels9050428 (PMC10218152; doi:10.3390/gels9050428)
Supplement: Supplementary file 1 [file gels-09-00428-s001.zip › Revised Supporting information 1.pdf]

Supporting Figures

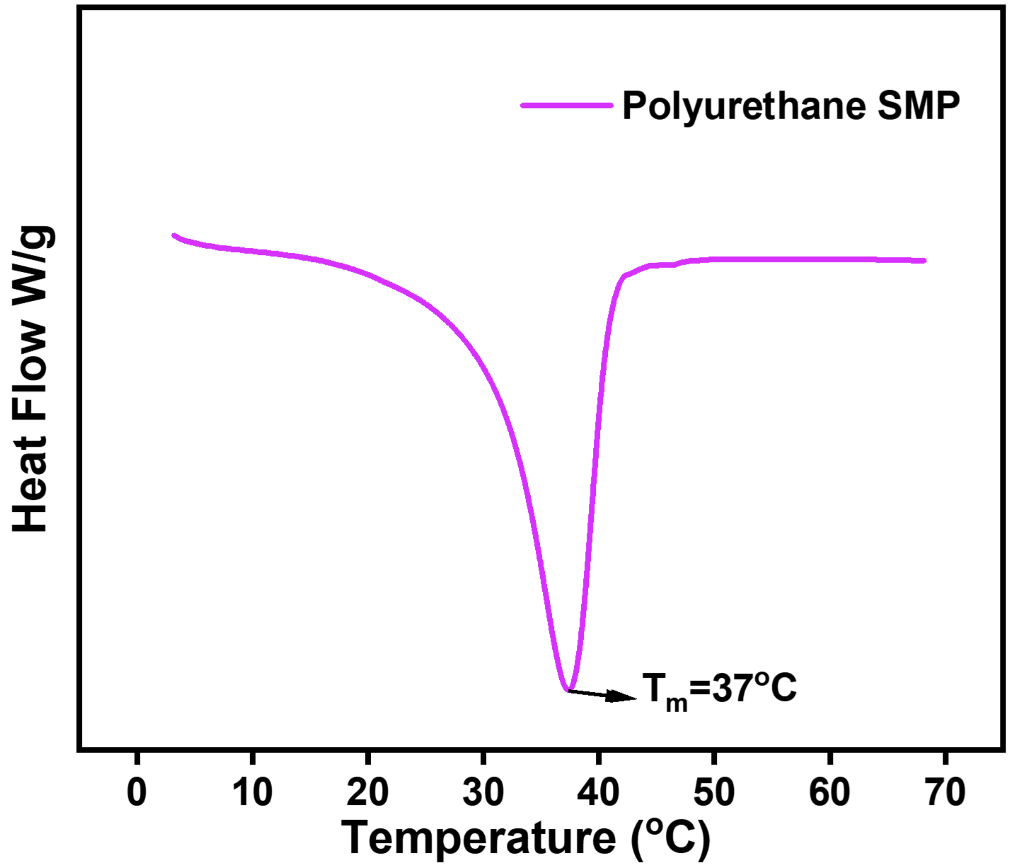

Figure S1. The DSC curve of the polyurethane SMP.

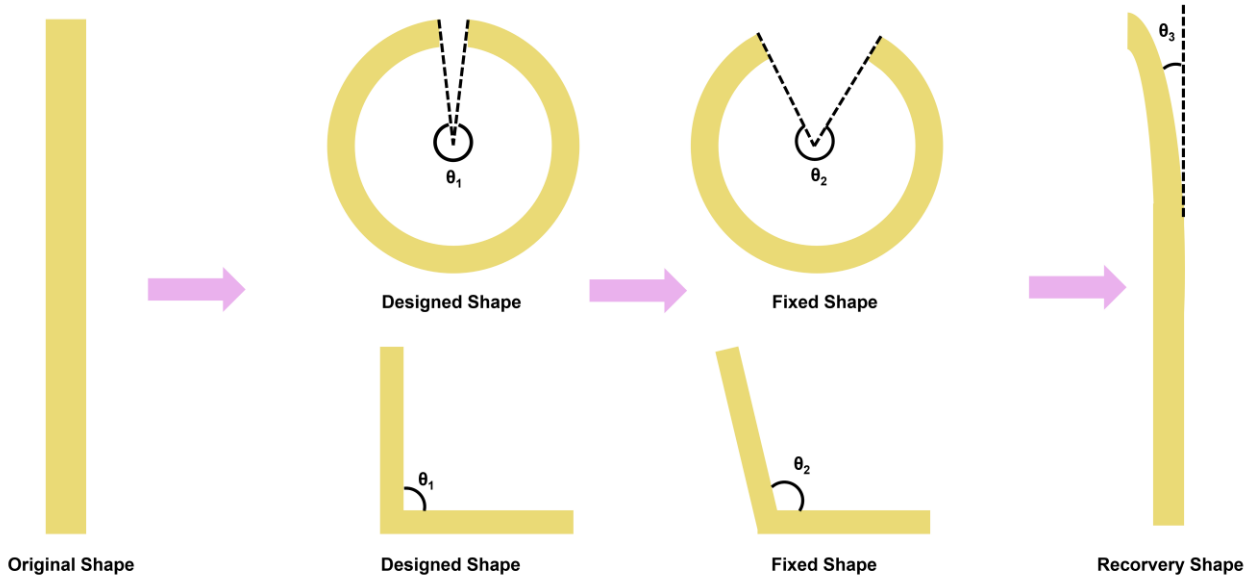

Figure S2. The angels of the polyurethane SMP under designed shape and fixed shape.

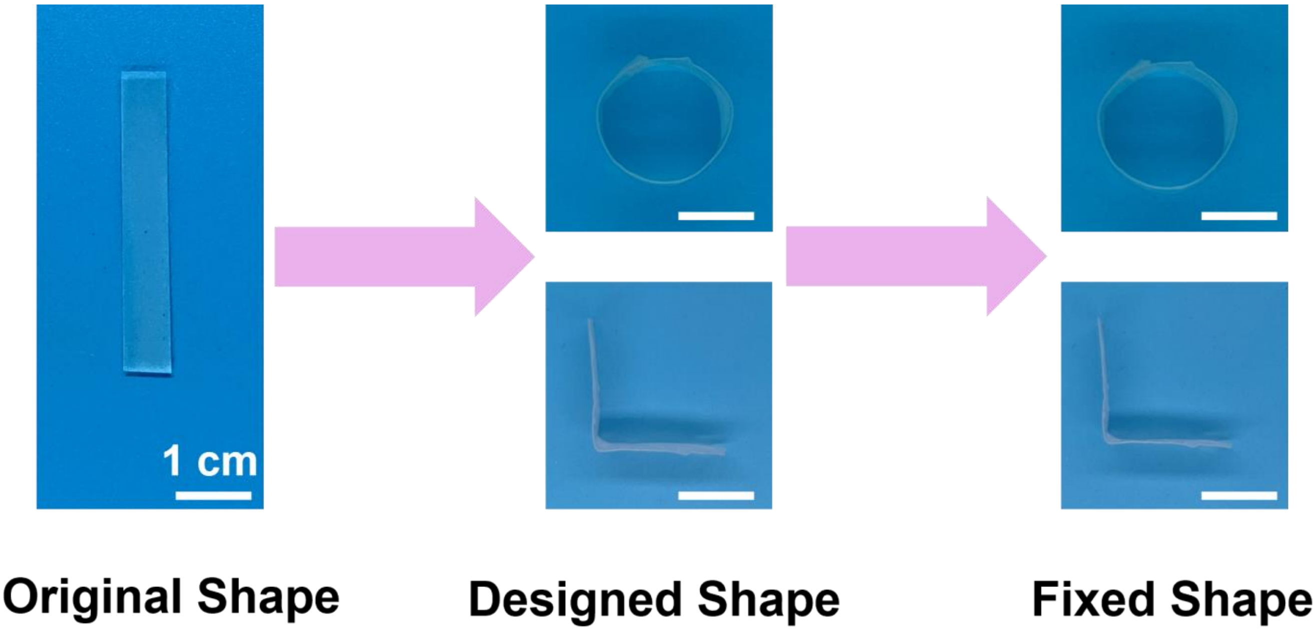

Figure S3. The angels of the polyurethane SMP under designed shape and fixed shape.

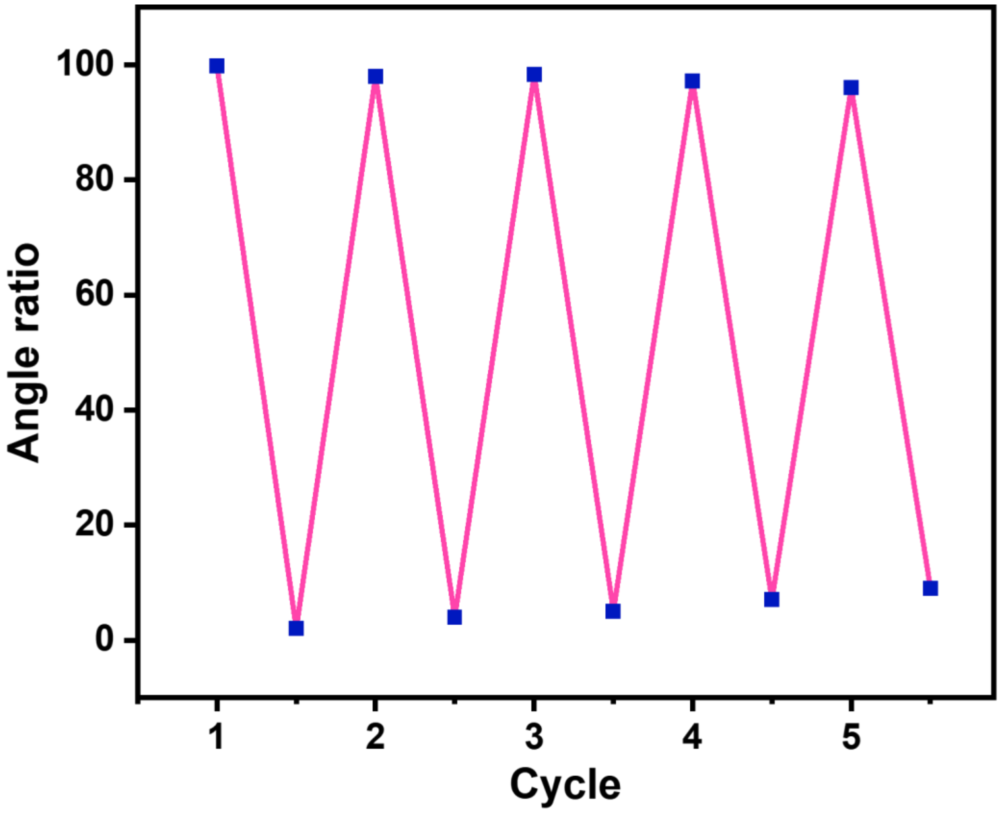

Figure S4. The cycle test of the polyurethane SMP.

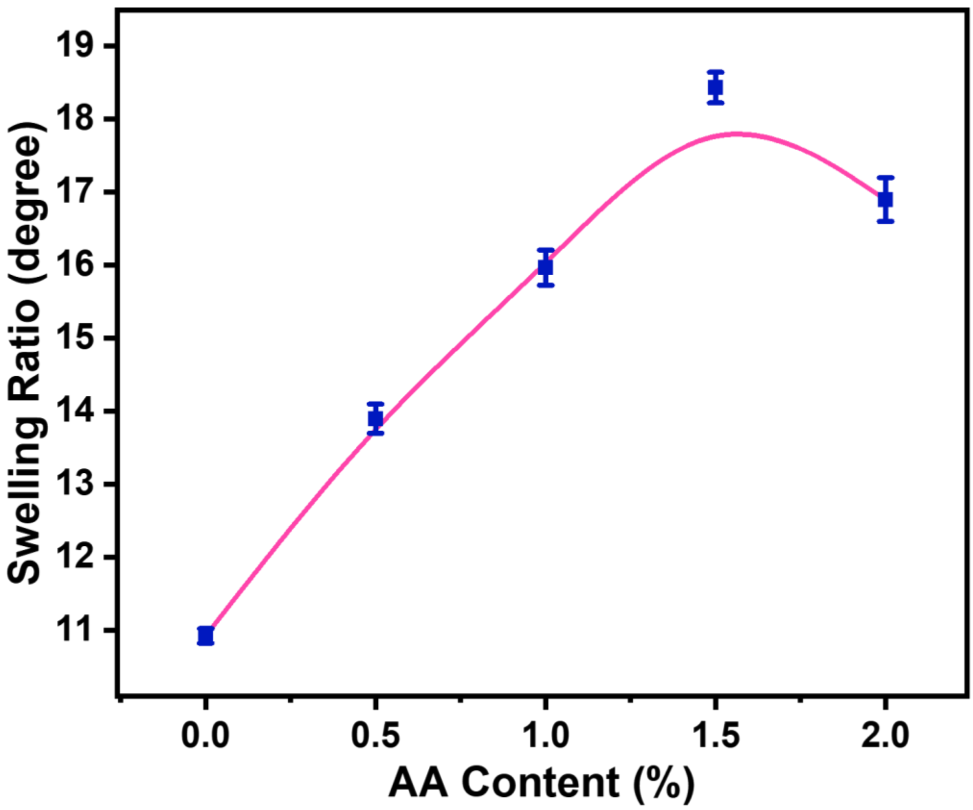

Figure S5. The swelling ratio of the PAA hydrogel with different content SMA in pH = 11 buffer solutions.

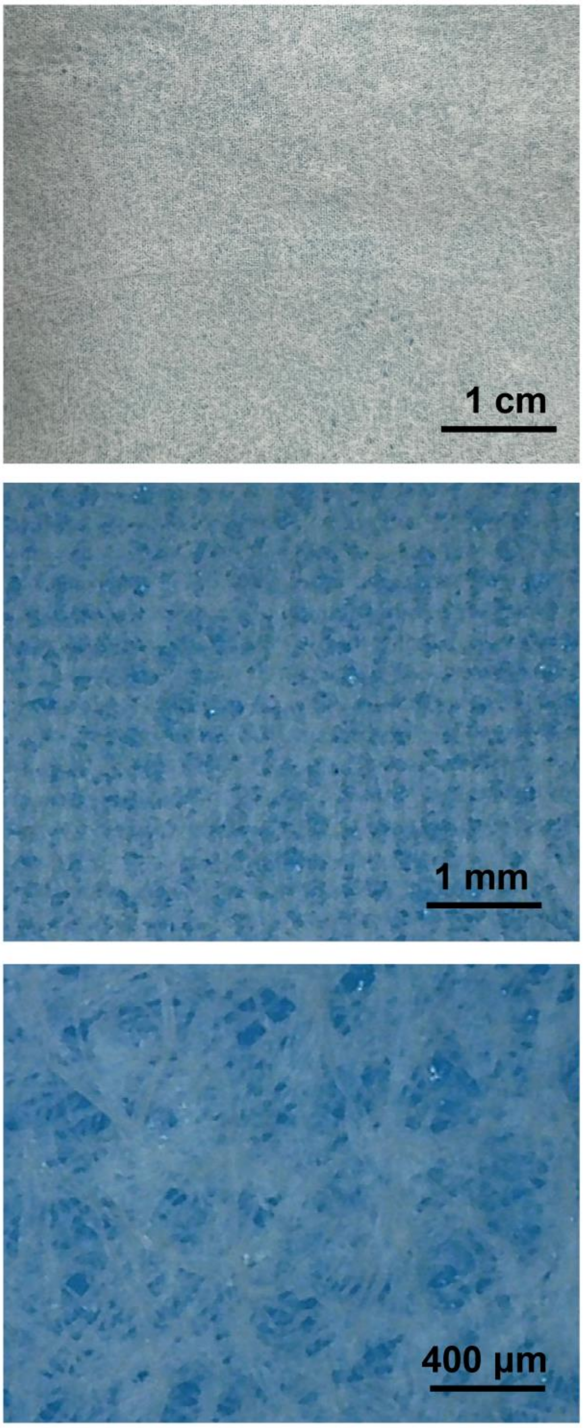

Figure S6. The optional images of cellulose-fiber based napkin.

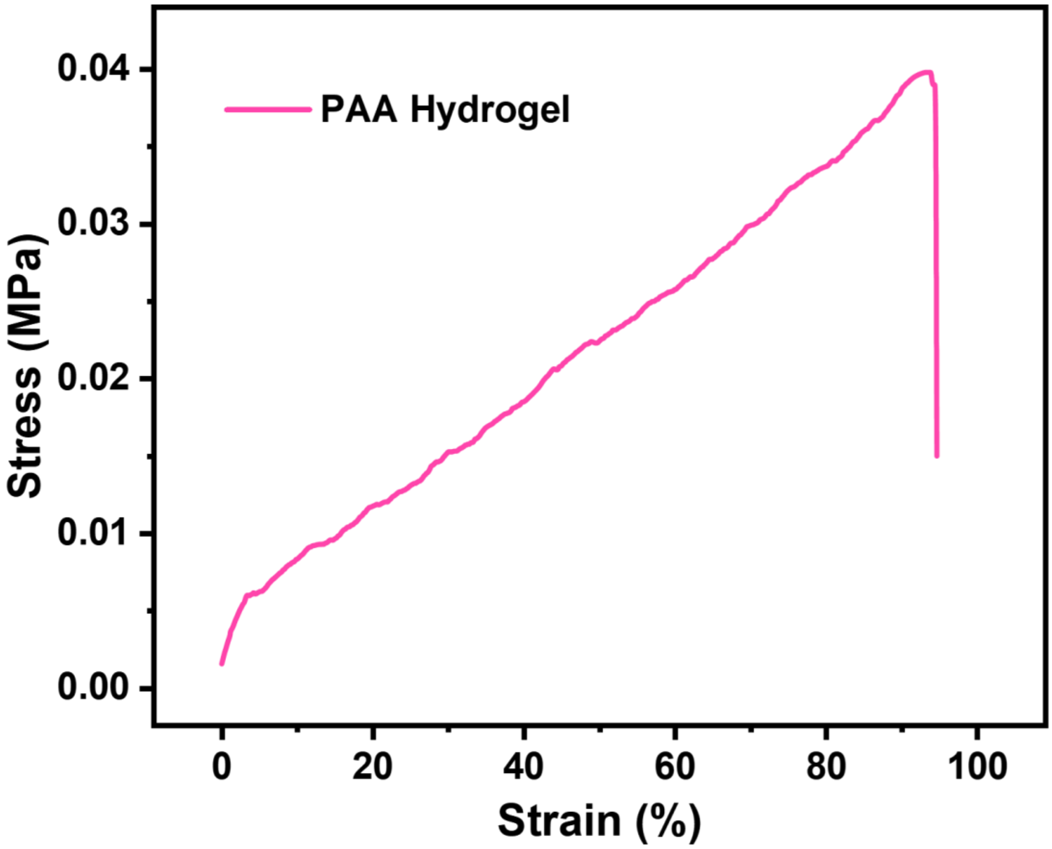

Figure S7. The mechanical property of the PAA hydrogel.

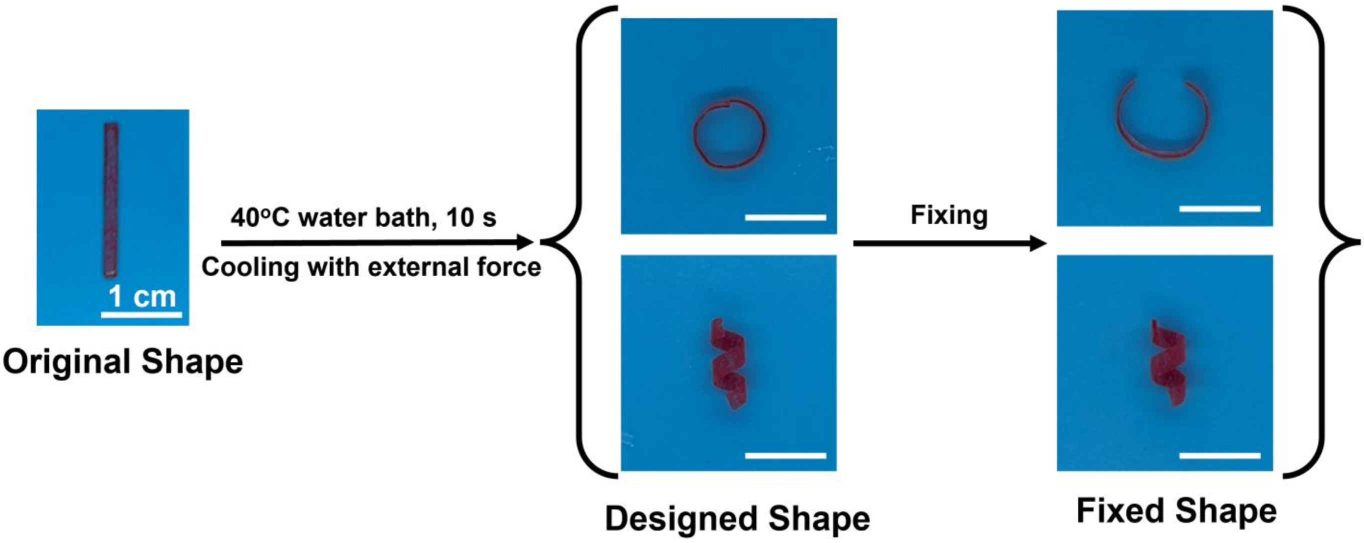

Figure S8. The Angles comparison of the actuator before and after swelling.

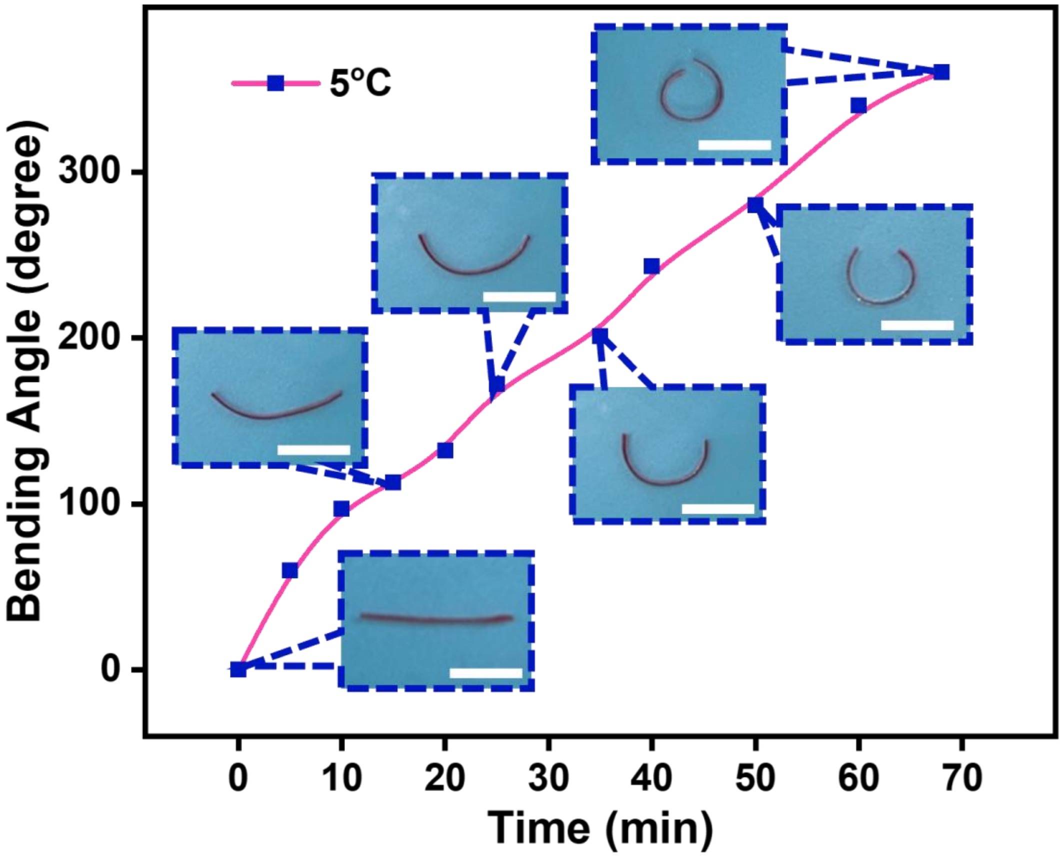

Figure S9: The actuation of the strip actuator in pH = 11 solution at 5°C. All scale bars are 10 mm.

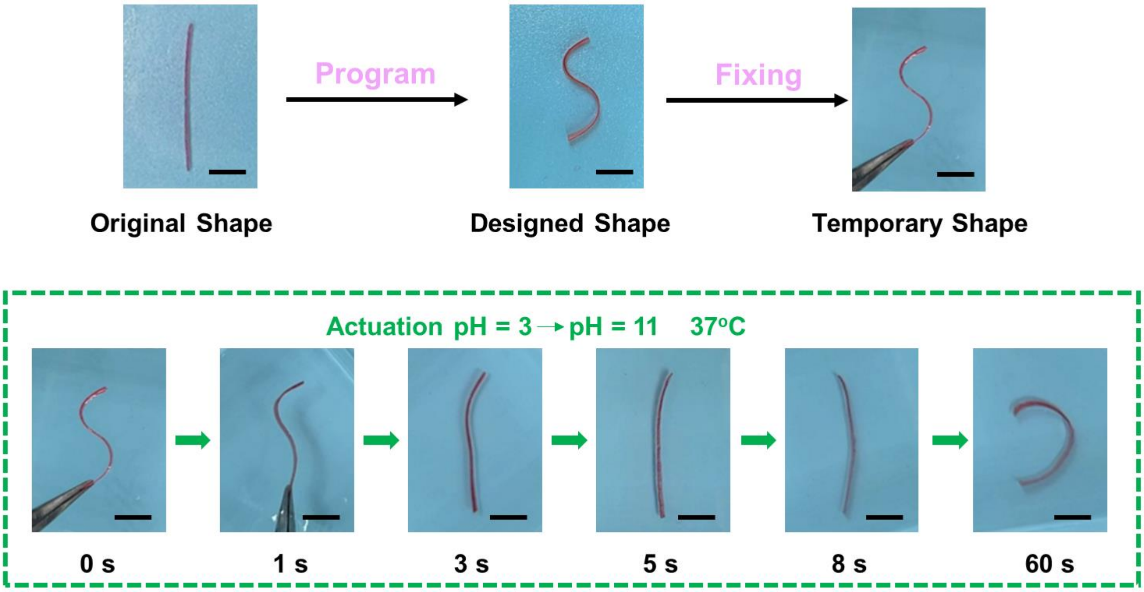

Figure S10: The actuation of the "S" shape actuator in pH = 11 solution at 37°C. All scale bars are 10 mm.

Supporting Movies

Movie S1. The actuation of the “S” shape actuator.

Movie S2. The actuation of the “M” shape actuator.

Movie S3. The actuation of the spiral shape actuator.

Movie S4. The standing process of the folding table.

Movie S5. The capturing process of the paw-mimetic actuator.

Movie S6. The jacking up process of the pangolin-mimetic actuator.

Movie S7. The hunting process of the octopus-mimetic actuator.
